# Monitoring the Efficacy of Oncolytic Viruses *via* Gene Expression

**DOI:** 10.3389/fonc.2017.00264

**Published:** 2017-11-06

**Authors:** Ashley Ansel, Joshua P. Rosenzweig, Philip D. Zisman, Beni Gesundheit

**Affiliations:** ^1^Rapo Yerapeh Ltd., Jerusalem, Israel

**Keywords:** oncolytic viruses, gene expression, oncolytic virotherapy, microarray analysis, transgenes

## Abstract

With the recent success of oncolytic viruses in clinical trials, efforts toward improved monitoring of the viruses and their mechanism have intensified. Four main gene expression strategies have been employed to date including: analyzing overall gene expression in tumor cells, looking at gene expression of a few specific genes in the tumor cells, focusing on gene expression of specific transgenes introduced into the virus, and following gene expression of certain viral genes. Each strategy presents certain advantages and disadvantages over the others. Various methods to organize the dysregulated genes into clusters have provided a window into the mechanism of action for these viruses. Methodologically, the combined approach of looking at both overall gene expression, the tumor cells and gene expression of viral genes, enables researchers to assess correlation between the introduction of the virus and the changes in the tumor. This would seem to be the most productive approach for future studies, providing much information on mechanism and timing.

## Introduction

With the development of recombinant deoxyribonucleic acid (DNA) technology in the early 1990s, the possibility of genetically engineering oncolytic viruses to improve virotherapy became a reality (1). Capitalizing on what was known about the mechanisms for oncolytic viruses, research focused on improving the safety profile, attenuating direct tumor lysis, and modulating the immune response. Beyond its potential therapeutic benefits, genetic engineering has generated new ways of monitoring and predicting sensitivity or resistance to oncolytic virotherapy (2). Various strategies, including animal models, cell lines, and even human *in vivo* gene expression studies, have been implemented to assess the effects of the oncolytic virus in a personalized way.

In 2015, the Food and Drug Administration approved the first oncolytic virus based on the results of a phase 3 trial for melanoma (3–5). Many other viruses are now being tested in clinical trials for various indications. This necessitates careful consideration of endpoints and creative new ways of monitoring therapeutic success.

Four main strategies for monitoring oncolytic viruses were surveyed: overall gene expression in tumor cells, gene expression of a few specific genes in the tumor cells, gene expression of specific transgenes introduced into the virus, and gene expression of certain viral genes.

### Monitoring Overall Gene Expression of Tumor Cells (See Tables 1–3)

**Table 1:** Tumor Genes.

Reference	Harvest time point: postinfection/other notes	Top upregulated genes	Top downregulated genes	Pathways/functional groups most affected
Haddad et al. (12)	6 h	SLC5A5, HIST2H4A, AK026847, HIST1H4E, HIST1H4B	BHLHB2, CX3CL1, G0S2, SOCS1	HMGB-1, interleukin (IL)-2, IL-6, IL8, Janus kinase/signal transducer and activator of transcription (JAK/STAT), interferon (IFN), and ERK 5 signaling
	
	24 h	SLC5A5, AK026847, HSPA6, HIST2H4A	IL8, ICAM1, SFRP1, CCL20, RSU1	P53- and Myc-induced apoptotic processes, pancreatic adenocarcinoma signaling, and phosphoinositide 3-kinase/v-ask murine thymoma vial oncogene homolog 1 (PI3/AKT) pathways

Balogh et al. (8)	12 h	Rsad2, Cxcl11, 10869879, Ddx60, Ifnb, Ifih1, Ifnb2, 10720237, Isg15, Herc6, Usp18, Oasl2, Oasl, Oas1b, Gbp5, Gbp1, Mx1, Irgm, Ifit1, Ifit2, Ifit3, Ifi47, Cxcl9, TRAIL, Tnf, Atf3	Tradd, Fadd, etnk2, trpc3, p2ry12, galr2, rpa3	Toll-like receptor signaling, RIG-I-like receptor signaling, IFN signaling, IFN effector pathways, apoptosis pathways, endoplasmic reticulum stress pathways, and cell cycle regulation

Lee et al. (15)	0, 24, 48, and 72 h	LEF1, PVRIG, SLFN11, LPP, CECR1, ARHGEF6, IRX3, STAMBPL1, IGFBP2, CD1D	CD151, AHNAK, TRIP6, LGALS1, MGST1, SRGN, CCND2, CCDC50, ITGB7, PDLIM1	Phosphoprotein, mutagenesis site, regulation of programmed cell death, lysosome, regulation of apoptosis, and surface antigen

Lacroix et al. (11)	72 h	EFTUD1, MMP1, PPM1F, LAMB3, TMEM200C, SIRPA, THEG, VPS18, RBM22, FOLR2, COX17, TFPI2, ACTL8	ZIC1, FLRT3, MYC, FOXG1, MAPT, NFIA, PHLPP1, ZNF671, FZD3	Steroid biosynthesis, ether lipid metabolism, TGF-beta signaling pathway, Wnt signaling pathway, gonadotropin releasing hormone signaling pathway, and the Jak-STAT signaling pathway

Reinboth et al. (29)	Early (2 h)	114 human genes strongly correlating with viral genes	Networks: posttranslational modification, free radical scavenging, gene expression, cell death, and cellular growth and proliferation. Molecular functions: cell cycle, cellular movement, development, growth and proliferation, and cell-to-cell signaling	
	
	Intermediate/late (10 h)	84 human (early) genes strongly correlating with viral genes (intermediate/late)	Cell death, cell cycle, lipid metabolism, small molecule biochemistry, and cellular development	
	
	48 h			Cell death, cellular growth and proliferation, protein synthesis and folding, infectious disease, genetic disorder, cell cycle, and deoxyribonucleic acid replication, recombination, and repair

Kurozumi et al. (6)	3 days	Cxcl11, Ifnγ, Cxcl9, Ccl12_predicted, Cxcl10, Ccl4, Il1b, Ccl5, Ccr6, Cxcr3	Spp1, Il6st	

Pfankuche et al. (9)	1 day	DDX60, DLA-79, CXCR7, F13A1, LOC100685890, CCR5, TRIM22, LOC100686473, GPR34, ENPEP	SERPINB2, TPM2, SCIN, VEGFB, THBS2, COL4A1, DMD, S100P, LOC608476, GSTA3	WebGestalt (UP): immune response-activating signal transduction activation of immune response; immune response-regulating signaling pathway; positive regulation of immune response; response to other organism; regulation of immune response; positive regulation of immune system process; regulation of immune system process; immune response; immune system process DAVID (UP): activation of innate immune response; cell migration; leukocyte proliferation; positive regulation of programmed cell death; positive regulation of leukocyte activation; regulation of leukocyte proliferation; blood coagulation
				WebGestalt (DOWN): blood vessel morphogenesis; positive regulation of cell migration; positive regulation of cell motility; cardiovascular system development; positive regulation of cellular component movement; circulatory system development; regulation of cell adhesion; positive regulation of locomotion; localization of cell; biological adhesion DAVID (DOWN): blood vessel development; protein amino acid glycosylation; organic acid metabolic process; regulation of neurological system process; regulation of transferase activity; blood coagulation; nucleobase, nucleoside and nucleotide metabolic process; antigen receptor-mediated signaling pathway; leukocyte proliferation

Josupeit et al. (14)	Most significantly expressed in susceptible cells	FAM49B, B4GALNT1, COL4A5, SLITRK4, SLC26A10, IFITM3, ASAP1, LAYN, NTRK2, ARHGEF25, CTGF, NXPH1, UGT8, NCAN, NAP1L3		
	
	Most significantly expressed in resistant cells	CTHRC1, RPS4Y1, EIF1AY, DDX3Y, DPYD, PNMAL1, S100A10, TXLNG2P, TRIM38, SPP1, KDELR3, SPARCL1, MPPED2, FABP6, CCDC71L, EDNRB, TSPAN31, FAM213A		

Garcia et al. (30)	0, 2, 6, 12, 18, 24, 30, 36, and 48 h postinfection	TNFAIP8		

Tanaka et al. (16)	0, 6, 24, and 48 h postinfection	SAMD9		

Kurozumi et al. (17)	12 h postinfection	CYR61, Ang-2	TSP-1	

Zhang et al. (7)	3 and 6 weeks (from the mouse chip microarray)	Ly6a, Plac8, Ly6c, Ccl8, Ifitm3, Ms4a4c, Clec4e, Ly6e, Tgtp, Ifit1, Rsad2, Ccl2, Ifi27, Ifi47, Ccl7, Dck, Ifit3, Irf7, Gas1, Gbp2, Cd69, Il18	Elavl2, Lmcd1, Arr3, Trip4, Crmp1, Hpd, Ewsr1, Ociad2, Cox15, Hmgn3, Nfia, Cables1, Rfxank, Tusc4, Cnot3, Magi1, Mrg2, Stag1, Sca2, Pdcd2, Tub, Ndrg1, Pigl	UP: major histocompatibility class I, chemokine receptor binding, chemokine activity, and cytokine activity; down: peptidases, proteases

Jiang et al. (22)	24 h	Tumor necrosis factor		

Li et al. (20)	48 h		MYCN	

Ma et al. (19)	3 days	Dm-dNK		

Saito et al. (10)	14, 18, and 22 h	CASP3, CASP7, CASP8, CASP9, CASP10, CYCS, IKK, NF-κB		

Han et al. (18)	12, 24, and 48 h	*Staphylococcus* enterotoxin A		

**Table 2:** Methods.

Reference	Virus type	Virus name	Type of samples	Gene expression analysis	Viral analysis	Pathway analysis
Haddad et al. (12)	Vaccinia	GLV-1h153	Human pancreatic cancer cells	HG-U133A cDNA microarray chips	Green fluorescent protein (GFP) expression	Ingenuity Pathways Analysis (IPA)
Balogh et al. (8)	Newcastle disease virus	MTH-68/H	Rat adrenal tumor cells	Affymetrix exon chip/microarray, quantitative reverse transcriptase PCR		DAVID functional annotation-clustering tool
Lee et al. (15)	Vaccinia	Pexa-Vec	Human hematologic malignant cells	Microarray	qPCR	DAVID functional annotation-clustering tool
Lacroix et al. (11)	Oncolytic parvovirus	H-1PV	Human medulloblastoma cells	Microarrays, quantitative real-time PCR (QRT-PCR)	QPCR-assay, dot blot assay	KEGG pathway analysis
Reinboth et al. (29)	Vaccinia virus (VACV)	GLV-1h68	Human melanoma cell lines	Microarray	Customized Affymetrix platform, GFP expression	IPA software
Kurozumi et al. (6)	HSV-1	hrR3	Implanted Rat glioma cells intracranially into immune competent rats	Quantitative real-time polymerase chain reaction-based microarrays, enzyme-linked immunosorbent assay (ELISA) for interferon-gamma expression by ELISA		
Alain et al. (25)	Reovirus	Dearing strain of reovirus serotype 3	Human glioma cells and Ras mouse embryo NIH3T3 cells	Northern blotting	Immunofluorescence	
Carey et al. (13)	Vesicular stomatitis virus (VSV)		Human LNCaP and PC3 cells	Real-time reverse transcription (RT)-PCR, microarray analysis		IPA software
Gholami et al. (27)	Vaccinia	GLV1h-153	Human triple negative breast cancer cell lines		GFP expression	
Pfankuche et al. (9)	Canine distemper virus (CDV)	DH82-Ond-pi	Canine histiocytic sarcoma cell line and *in vivo* SCID mice model	Microarrays	Immunofluorescence	WebGestalt and DAVID
Josupeit et al. (14)	Oncolytic parvovirus	H-1PV	Human NCH421k cells and the NCH421R and NCH421I subclones	Affymetrix human genome-U133 plus 2.0 microarray	Dot blot assay, immunofluorescence	
Garcia et al. (30)	CDV		Human mammary tumor and canine-derived adenofibrosarcoma cell lines	Quantitative polymerase chain reaction (qPCR)	qPCR	
Tanaka et al. (16)	Inactivated Sendai virus particle	HVJ-E	Human glioblastoma cell line U251MG	Real-time quantitative PCR, microarrays		
Hirvinen et al. (21)	VACV	vvdd-tdTomato-hDAI	Human melanoma HS294T and human monocyte THP-1 cells	Whole Genome sequencing	Fluorescence	BACA, David, and IPA analysis
Kurozumi et al. (17)	HSV-1	hrR3	Human U343, U87, U87ΔEGFR, and LN229 glioma cell lines, rat glioma D74/HveC cells, Fischer rats 8–10 weeks of age, and athymic nude mice 6–8 weeks of age	QRT-PCR		
Zhang et al. (7)	VACV	GLV-1h68	Human ductal adenocarcinoma GI-101A cells were injected into 6- to 8-week-old female, nude mice	GeneChip mouse genome array and human genome U133 plus 2.0 array	GFP and fluorescence microscopy	Gene ontology (GO)
Jiang et al. (22)	Adenovirus	SG502-TNF	Human A549 lung cancer cell line and human TE-1 esophageal cancer cell line	SYBR green I PCR	GFP and fluorescence	
Li et al. (20)	Adenovirus	ZD55-shMYCN	LA1-55N human neuroblastoma cell line	QRT-PCR		
Ma et al. (19)	Adenovirus	ZD55-Dm-dNK	HCT-116 and SW620 Human colorectal cancer cell lines	RT-PCR and enzyme assay	Western blot analysis	
Saito et al. (10)	Sindbis virus	SIN AR399	HSC-3 and HSC-4 human oral squamous cell carcinoma cell lines	Real-time quantitative RT-PCR	Viral titers, immunoblot analysis	
Han et al. (18)	Adenovirus	PPE3-SEA	MB49 mouse bladder cancer cells	RT-PCR	Western blot analysis	
Sato et al. (26)	Adenovirus	OBP-301 and OBP-401	Acc2 and AccM human salivary gland adenoid cystic carcinoma cell lines	Quantitative real-time RT-PCR analysis (viral gene)	GFP and fluorescence	
Guse et al. (24)	Adenovirus	Ad5/3-Δ24, Ad5-Δ24pK7, Ad5-Δ24RGD, Ad5-Δ24E3, Ad300wt, Ad5LacZ	HEY human ovarian cancer cells, 786-O human renal cancer cells, and 4- to 5-week-old female nude mice		Real-time quantitative PCR was done with a SYBR green assay using a RotorGene system and fluorescence	
Shin et al. (23)	VSV	rVSV-IL12, rVSV-F	SCC 09 and FaDu human squamous cell carcinoma cell lines, and SCC VII murine squamous cell carcinoma cell line, and 6-week-old female C3H/HeJ mice	Real-time reverse transcriptase-polymerase chain reaction assays		

**Table 3:** Gene Overlap.

Upregulated genes	Reference	Gene overlap by papers				
		Downregulated genes	Reference	Mixed genes	Upregulated	Downregulated
Rsad2	Balogh et al. (8), Zhang et al. (7)	MYC/MYCN	Lacroix et al. (11)/Li et al. (20)	SPP1	Josupeit et al. (14)—resistant	Kurozumi et al. (6)
Cxcl11	Balogh et al. (8), Kurozumi et al. (6)	NFIA	Lacroix et al. (11), Zhang et al. (7)			
Ddx60	Balogh et al. (8), Pfankuche et al. (9)					
Ifit1	Balogh et al. (8), Zhang et al. (7)					
Ifit3	Balogh et al. (8), Zhang et al. (7)					
Ifi47	Balogh et al. (8), Zhang et al. (7)					
Cxcl9	Balogh et al. (8), Kurozumi et al. (6)					
TNF	Balogh et al. (8), Jiang et al. (22)					
IFITM3	Josupeit et al. (14)—susceptible, Zhang et al. (7)					

The first method of assessing the impact of oncolytic virotherapy involves monitoring overall gene expression of tumor cells before and after virotherapy. To identify genes whose expression was altered in tumor cells during infection with an oncolytic virus, genome-wide expression profiling needs to be performed. The studies surveyed included both animal studies and studies performed on human cell lines. Kurozumi et al. used a rat intracranial glioma model in immune competent rats (6). They introduced a type one herpes simplex virus (HSV-1) and used quantitative real-time polymerase chain reaction (PCR)-based microarrays to monitor tumor gene expression. They found that oncolytic virus treatment induced at least a twofold increase or decrease in the expression of 50 genes when compared with the control. More specifically, they found numerous genes were upregulated from the chemokine family (see Table 1). Unfortunately, Kurozumi et al. did not analyze gene clusters for these genes. Similarly, Zhang et al. also looked at tumor gene expression using an animal model. In this case, they used nude mice injected with metastatic human breast adenocarcinoma GI-101A cells (7). They monitored tumor gene expression after treatment with a vaccinia virus (VACV) using a mouse genome array and a human genome array. They found 681 genes differentially expressed when compared with controls. As opposed to the Kurozumi group, Zhang et al. used gene ontology to evaluate clustering and found upregulated genes related to the immune system, and downregulated genes related to enzymatic function (see Table 1).

However, some reservations must be expressed regarding the use of Affymetrix mouse arrays. Since they are relatively species specific, genes that are identified as differentially expressed may primarily represent host cells infiltrating the tumor. When assessing the changes of potential cross hybridization of human genes to the mouse chip, less than 50% of the genes identified with the mouse arrays were differentially expressed according to the human chip. Furthermore, the immune-related genes that were differentially expressed displayed very low fluorescence intensities in human as compared with very high intensities displayed by the same genes in the mouse array. This would suggest that perhaps comparisons of gene expression between species should be evaluated with caution.

Balogh et al. used a rat adrenal tumor cell line, namely, pheochromocytoma cells, as the model, and looked at gene expression after infection with a recombinant oncolytic viral strain of Newcastle disease virus called MTH-68/H (8). They used a rat specific microarray chip to monitor gene expression and confirmed changes *via* quantitative reverse transcriptase PCR. They found that 729 genes were upregulated and 612 genes were downregulated with oncolytic viral treatment compared with controls. Balogh et al. relied on DAVID functional annotation-clustering tool to group the genes according to function. This clustering tool was far more elaborate than the method used by Zhang. They found pathways including receptor signaling, apoptosis, and cellular stress to be involved (see Table 1). The detailed report they provided was extremely helpful in beginning to understand the mechanisms involved in oncolytic viral therapy. In addition, Pfankuche et al. used a canine sarcoma cell line as the tumor model and injected a canine distemper virus (CDV) called DH82-Ond-pi (9). They visualized the cells using immunofluorescence and used a canine specific microarray chip to evaluate tumor gene expression. They identified 892 upregulated genes and 869 downregulated genes when compared with controls. They analyzed these results using WebGestalt and DAVID and found that upregulated genes were primarily related to immune processes, cell migration, apoptosis, and blood coagulation (see Table 1). Certainly, the role of oncolytic viruses in impairing angiogenesis demands further attention based on the mechanism related clues provided by this gene expression study.

Beyond the studies looking at animal cell lines, there are a few studies that looked at tumor gene expression in human cell lines. Saito et al. used two human oral squamous cell carcinoma cell lines called tHSC-4 and HSC-3 and injected the Sindbis SIN AR399 oncolytic virus (10). Using real-time quantitative reverse transcription (RT)-PCR to monitor the tumor gene expression after treatment with the virus, they found that Caspases 7, 8, and 10 were upregulated in both HSC-3 and HSC-4 cells, but that Caspases 3 and 9, cytochrome *c*, NF-κB, and IKK were only upregulated in HSC-3 cells. The likely interpretation of these data is that SIN induced oncolysis in HASC-3 cells by activating a few apoptotic pathways. Similarly, Lacroix et al. looked at six human medulloblastoma cell lines (MB) and how they are affected by treatment with the oncolytic parvovirus H-1 (H-1PV) (11). They used microarray and quantitative real-time PCR (QRT-PCR) to evaluate gene expression after oncolytic virus treatment. They focused on the 25 most significantly upregulated and the 25 most significantly downregulated genes. They used KEGG pathway analysis to identify clusters of genes and found that five pathways were particularly impacted by H-1PV infection. These included the pathways for steroid biosynthesis, ether lipid metabolism, TGF-beta signaling, Wnt signaling, gonadotropin releasing hormone signaling, and Jak-STAT signaling. One advantage in the methodology employed by the Lacroix group was focusing on the top 25. As opposed to the other groups that tried to find patterns from all the up and downregulated genes, Lacroix et al. potentially eliminated noise and were able to better identify the most important pathways. Finally, Haddad et al. looked at the PANC-1 human pancreatic cancer cell line infected with GLV-1h153, an oncolytic VACV (12). They used cDNA microarray chips to monitor gene expression after infection with the virus and used a cutoff of a twofold change to identify the most relevant genes. At 6 h postinfection, they found that 139 genes were up- or downregulated, but by 24 h after infection 5,698 genes were dysregulated. They analyzed the pathways using the Ingenuity Pathways Analysis (IPA) software and found that downregulated genes clustered around pathways associated with cell death, cell cycle, and DNA repair. Upregulated genes were associated with mechanisms related to infection. In contrast to the groups surveyed so far, Haddad et al. also labeled the virus with green fluorescent protein (GFP) and used this to determine if GFP-marker gene expression can be correlated with viral copy number. Analyzing GFP expression levels in the cells infected by the virus was shown to be both time and multiplicity of infection dependent. Considering that by 24 h postinfection almost 70% of live cells expressed GFP, and that the amount of dysregulated genes was significantly higher by 24 h, it seems reasonable to assume that the virus significantly affected the tumor. The pathway analysis enabled Haddad et al. to go a step further and begin to understand the method by which the viruses affect the tumor cells. More robust examples of combined testing of both tumor gene expression and viral levels will be reviewed in a later section.

The human studies reviewed until this point compared between tumor cells and normal cells. A slightly different methodology was employed in a few studies in which gene expression was compared between susceptible and resistant cancer cells. For example, Carey et al. capitalized on the fact that vesicular stomatitis virus (VSV) replicates selectively in cancer cells that have defects in the interferon (IFN)-I pathway (13). They looked at two different lines of prostate cancer cells. The first, human LNCaP cells, possess a defective IFN-I response, making them sensitive to VSV infection. The second, human PC3 prostate cancer cells, on the other hand, have functional IFN-I signaling, making them resistant to VSV infection. They employed real-time RT-PCR analysis and found that primary transcription, secondary transcription, and viral protein synthesis were delayed in PC3 cells compared with LNCaP cells. To look at gene expression, they used microarray and found that PC3 cells expressed many antiviral gene products compared with LNCaP cells. Furthermore, they looked at 80 different signaling pathways using IPA software and found specific pathways to be associated with a difference in gene expression between the two cell lines. Predictably, the IFN pathway had the highest percentage of differentially expressed genes. This research suggests the possibility of sensitivity markers for VSV treatment and hints at the mechanism of action of the virus. Similarly, Josupeit et al. looked at human NCH421k glioblastoma multiforme cells, which are susceptible to infection by parvovirus H-1 (H-1PV) and compared its response to H-1PV with NCH421R cells, which are a subclone resistant to H-1PV (14). They used “stem like” cell lines in NOD/SCID mice. They found a decrease in metabolic activity in the sensitive cell line compared with the resistant cell line when treated with H-1PV. When they analyzed gene expression using the Affymetrix Human Genome-U133 plus 2.0 microarray, they found 201 genes that were differentially expressed by at least threefold. They used unsupervised clustering to group the differentially expressed genes into three different categories. Some of these gene products are involved in regulating the antiviral immune response. A further example of comparing the response to oncolytic viruses in susceptible and non-susceptible cell lines is the study done by Lee et al. They compared the response of sensitive human myeloid leukemia lines and resistant human lymphoid leukemia cell lines to Pexa-Vec, a VACV engineered to express human granulocyte-macrophage colony-stimulating factor and β-galactosidase (15). Using quantitative PCR (qPCR) they found that 660 genes were upregulated at least twofold and 776 genes were downregulated at least twofold in the lymphoid cancer cell lines. In the case of the upregulated genes, changes were particularly remarkable, with more than 50 genes induced fivefold or higher, and 150 genes that were expressed three or fourfold higher, than the control. They used the DAVID functional annotation-clustering tool to classify the genes into 319 functional gene clusters. Some of the clusters included genes related to: viral replication and regulation of apoptosis.

### Gene Expression of Specific Genes in the Tumor Cells

A second strategy currently employed to monitor the efficacy of oncolytic virotherapy is to monitor the expression of specific genes in the tumor cells. Interestingly, to the best of our knowledge, no such human studies have been published. However, a number of human cell-line studies have been done. For example, Tanaka et al. focused on the sterile alpha motif containing domain (SAMD9) gene in the human glioblastoma cell line called U251MG (16). They treated this line with Sendai virus particle (HVJ-E). Using real-time quantitative PCR and microarray analysis they found that SAMD9 gene was upregulated in tumor cells treated with the virus and the SAMD9 messenger ribonucleic acid (mRNA) was upregulated in a time and dose-dependent manner. In a study by Kurozumi et al., they also looked at a limited number of genes in the tumor cells, but they looked at both human and animal cell lines (17). They looked at 10 genes in glioma cell lines following treatment with HSV-1 virus hrR3 and compared the response to controls. When they used QRT-PCR, they found that three genes in particular were dysregulated. The first, the antiangiogenic factor *TSP-1* was downregulated, and the other two, angiogenic factors *CYR61* and *Ang-2* were significantly upregulated when compared with controls. The advantage of this study over the previous one is that they were able to correlate the upregulation of CYR61 gene expression with the presence of the virus in the tumor tissue *in vivo*. CYR61 is a known protein that is involved in apoptosis, angiogenesis, the cell cycle, and extracellular matrix formation. By focusing on known genes but looking at the tumor cell expression in response to viral therapy and looking at the presence of the virus, researchers are able to better pinpoint the mechanisms at work when a virus infects a tumor cell.

### Monitoring Transgene Expression

A third and particularly innovative strategy currently employed to monitor the efficacy of oncolytic virotherapy is the introduction of transgenes into the oncolytic virus and then analyzing gene expression of the transgene specifically. As we saw with the specific gene studies, there were no known published human studies. However, we will analyze one animal line study and a number of studies looking at human cell lines. Han et al. developed an oncolytic adenovirus PPE3-SEA that expressed the superantigen *Staphylococcus* enterotoxin A (SEA) and that has improved tumor specificity due to regulating the expression of E1A and E1B genes (18). They tested the PPE3-SEA virus against MB49 mouse bladder cancer cells *in vitro* and *in vivo*. They found that the mice treated with the virus had a significantly lower mean tumor volume than the control group. This seemed to correlate well with the increased expression of the virus mRNA *in vitro*.

In the human cell line studies, for example, Ma et al. constructed an adenoviral vector ZD55-Dm-dNK, containing the *Drosophila melanogaster* multisubstrate deoxyribonucleoside kinase—an important suicide gene (19). They looked at the expression of Dm-dNK human in colorectal cancer cells (HCT-116 and SW620) using RT-PCR. They found higher expression of the virus in the colorectal cancer cell lines, and lower levels of expression in the normal cell controls.

Similarly, Li et al. constructed ZD55-shMYCN, an oncolytic adenovirus ZD55 targeting the MYCN gene (20). They treated a p53-null and MYCN amplified human neuroblastoma cell line LA1-55N with the new virus. Using a two-step real-time reverse transcription (RT)-PCR procedure, they found that the virus selectively replicated and significantly downregulated the MYCN expression and that it was capable of effectively silencing the MYCN gene and inducing apoptosis in the tumor cells. Furthermore, they were able to demonstrate that the virus inhibited the growth of xenograft tumor *in vivo*.

In addition, Hirvinen et al. developed an oncolytic VACV that expressed intracellular pattern recognition receptor DNA-dependent activator of IFN− regulatory factors (DAI) to stimulate the innate immune system and to activate adaptive immune cells in the tumor (21). They tested this virus on two different human cell lines: human melanoma cells (HS294T) and human monocyte cells (THP-1). They used the BACA representing tools *via* the DAVID database to analyze the genes. They found that in the THP-1 cell line, there were a lot more upregulated genes than in the melanoma cell line. They used IPA and found that the most upregulated networks involved pathways connected to the activation of the immune system. More specifically, pathways related to dendritic cell growth, communication between innate and adaptive immune systems, and recognition of viruses were dysregulated. Significantly, they also found a sevenfold upregulation of *DAI* in both cell lines.

Finally, Jiang et al. developed a recombinant adenovirus using SG502 and tumor necrosis factor (TNF), yielding SG502-TNF (22). They looked at the effect of the virus on two different human cell lines: human A549 lung cancer cell line and human TE-1 esophageal cancer cell line. With the help of SYBR green I PCR, they found that the expression of TNF protein increased in both cell lines after infection with the virus. Furthermore, they found that the virus attacked the tumor cells specifically, and that they regulated the apoptotic-signaling pathway.

Shin et al. investigated human squamous cell carcinoma cell lines, murine squamous cell carcinoma cell lines, as well as a murine model (23). They used a VSV that was engineered to express the murine interleukin (IL) 12 gene called rVSV-IL12 and compared it to a non-cytokine carrying VSV virus called rVSV-F. They found that both viruses demonstrated similar infection efficiency. Real-time RT-PCR and enzyme-linked immunosorbent assay were used to look at viral replication and IL12 expression. They found that human squamous cell carcinoma cell lines infected with rVSV-IL12 had a high level of IL12 expression at 48 h postinfection. In the murine model, the animals treated with virus had a smaller tumor area than the control group. The mice treated with rVSV-IL12 had a much greater reduction of the tumor compared with the mice treated with rVSV-F. *In vivo* they showed that by day 30, none of the control mice survived, yet 3 animals injected with rVSV-F and 10 animals injected with rVSV-IL12, survived beyond day 30.

### Monitoring Viral Gene Expression (See Table 4)

**Table 4:** Viral Proteins Monitored.

Viral proteins monitored	
Reference	Protein
Alain et al. (25)	S1
Garcia et al. (30)	CDVM
Li et al. (20)	E1A
Sato et al. (26)	E1A
Guse et al. (24)	E1A

In assessing the impact of oncolytic virotherapy, a fourth method is to look at the gene expression of the virus itself inside of the tumor cells over time. By marking the virus with GFP and measuring the viral gene expression over time within the tumor cells, there is a clear indicator of viral growth, followed by a decrease in viral presence in the tumor cell as the tumor cell is destroyed. This method has been demonstrated in a number of studies including: animal studies (*in vivo*), and *in vitro* studies with cell lines, but no human studies have been published to date to the best of our knowledge.

#### Studies Where Human Cell Lines Implanted into Animals (*In Vivo*)

In conducting animal studies, human cell lines are implanted into animals (*in vivo*). For example, Guse et al. used two different murine xenograft models, one for renal cancer and a second for ovarian adenocarcinoma (24). They co-injected a luciferase-encoding virus with eight different adenoviruses. In the ovarian cancer cell model, they found using PCR that the mice infected with some of the adenoviruses had an over 3 log increase in luciferase gene expression, a luminescence gene, compared with mice infected with other adenoviruses. They also found that gene copies of luciferase genes were increased in some of the models and decreased in others. In the renal cancer model, they used qPCR to monitor gene copies and found that they increased by three orders of magnitude in some of the lines but not at all in others. Bioluminescence demonstrated photoemission for all of the tumors that were treated which implies that the virus entered the tumors.

#### Human and Mice Cell Line Studies (*In Vitro*)

Shifting to *in vitro* studies, Alain et al. looked at human glioma cells, Ras-transformed mouse embryo NIH3T3 cells, reovirus resistant human glioma cells, and untransformed NIH3t3 cells (25). They infected these four cell lines with mammalian orthoreoviruses. Using Northern blotting, they found that outer capsid protein sigma-1 reovirus transcripts were found only in the Ras-transformed cell line and the susceptible cell line. However, they found that when the cells were treated with an E64 protease inhibitor it successfully blocked the virus. Treating with an infectious subviral particle enabled the virus to be detected even in the resistant cell line and even in the presence of E64. Furthermore they found that the level of active cathepsin B and L was increased in tumors.

Sato et al. looked at human salivary gland adenoid cystic carcinoma cell lines (26). They infected the cells with a telomerase-specific replication-selective adenovirus (OBP-301) and OBP-401, a genetically engineered adenovirus with the GFP gene. Using quantitative real-time RT-PCR, they found that E1A expression increased in infected cells. When using the virus with GFP, the intensity of the fluorescence increased in a dose-dependent manner. Another *in vitro* study was conducted by Gholami et al. They used the human triple negative breast cancer cell lines HCC38 and MDA-MB-468 (27). They infected the cells with a VACV GLV-1h153 that was engineered to express the human sodium iodine symporter gene. Using GFP, they found that the virus infected the cell lines in a time-dependent way that was proportional to the concentration of the virus.

#### Using Marker Peptides to Monitor Viruses Instead of PCR

Peng et al. developed oncolytic viruses that could be tracked *via* marker peptides (28). They used the Edmonston vaccine strain of measles virus (MV-Edm) to express either human carcinoembryonic antigen (CEA) or beta-human chorionic gonadotropin (βhCG). They injected MV-shCEA or MV-βhCG into two groups of transgenic mice. They compared detection of CEA in serum to RT-PCR for nucleocapsid RNA and found that serum CEA was a more sensitive method than PCR.

### Combination Studies (Viral and Host Gene Expression)

Until now we have reviewed studies that primarily looked either at tumor gene expression or viral gene expression. Perhaps the most interesting studies are the studies that combine both. Combination studies can potentially assess the correlation between the viral replication and the host response. Reinboth et al. looked at two different human melanoma cell lines (888-MEL and 1936-MEL) (29). They infected the cell lines with an attenuated VACV GLV-1h68. They used a platform called 36K to monitor human gene expression and they used a customized platform to monitor viral expression. To monitor viral expression, various markers were used including RUC-GFP, gusA, and the viral IFN-α/β-receptor-like secreted glycoprotein. The levels of the first two markers increased after infection, and the glycoprotein was expressed exclusively by GLV-1h68. To analyze the relationship between host cell transcription and viral replication, they assessed the correlation between viral and human gene expression at 2 h postinfection (early) and at 10 h postinfection (intermediate/late). At 2 h postinfection, they found 7 VACV genes and which were correlated to 114 human genes. Analysis with IPA demonstrated that these genes were related to the following pathways: apoptosis and the cell cycle, posttranslational modification, cellular growth and signaling, and other networks (Table 1). They then assessed whether human early gene transcription was predictive of VACV intermediate/late transcription and found 84 human early genes that correlated. These genes were important in processes such as cellular development and death, and lipid metabolism amongst others. Upon looking at expression 48 h after infection (late), there was a significant change in the expression of genes related to cellular growth, cell death, protein synthesis and folding, DNA replication, and DNA repair.

Similarly, Garcia et al. used three human mammary tumor cell lines along with a cell line for adenofibrosarcoma of canine origin, and infected them with CDV (30). They were testing the sensitivity to CDV infection, cell proliferation, apoptosis, mitochondrial membrane potential and expression of tumor necrosis factor-alpha-induced protein 8 (TNFAIP8). Using qPCR they were able to quantify both TNFAIP8 gene, and the virus CDVM gene expression; they found that both TNFAIP8 and CDVM gene expression were positively correlated in all cell lines.

## Critical Analysis: Advantages/Disadvantages of Four Strategies

In critically assessing the advantages and limitations of each of the four strategies described earlier, various conclusions became clear. For example, when looking at overall gene expression changes of tumor cells, the advantages include that it generates the broadest view of what is happening inside the tumor cells after infection with the virus without the initial bias of inserting a gene and looking specifically at that gene. Of course, it is also provides a much wider scope which is more advantageous than looking at only one or two genes. The disadvantages of this method, on the other hand, is that they provide less specific information about specific pathways or mechanisms through which the tumor increases or decreases due to the wider lens used. In addition, this method provides no information on the viral gene expression or the correlation between the virus and the decrease in tumor size.

Upon analyzing the second method, namely, gene expression of specific genes in the tumor cells, we saw various strengths and weaknesses as well. For example, this method could potentially provide more information on the mechanism for tumor shrinkage or growth. In addition, since the method is more specific it makes it slightly easier to execute. Furthermore, once the specific genes are chosen, it enables a more in depth analysis of how these genes are important and how gene expression changes in the tumor cells after viral infection can have an impact on the cells.

On the other hand, looking at specific genes in the tumor cells might not reflect the overall tumor status since the broad picture of what is going on in the tumor cells is missing. Similar to the limitation of the first method, this method also does not provide any information on the viral gene expression or the correlation between the virus and the decrease in the tumor.

The third method of looking at transgenes is advantageous because it is a relatively simple study to do, allowing for effective monitoring of a specific transgene, and it is not “shooting in the dark” and looking for a wide variety of genes. In this way, researchers can test how effective a specific transgene performs. However, disadvantages include that it lacks the broader perspective of a study looking at overall gene expression and does not provide any indication of how the expression of genes in the tumor cells change after viral infection.

Monitoring viral gene expression is helpful in terms of providing information about how effectively the virus is infecting and replicating in the host cell, but provides no information about gene expression changes in the tumor cell or the mechanisms for tumor growth or shrinkage. Compared with analyzing marker peptides to monitor viral growth and replication, gene expression might not be as sensitive, but this requires further studies to confirm this finding.

Overall, in terms of future directions, it would seem that combination studies are the optimal method for studying gene expression changes. They potentially allow both a broad picture of gene expression changes in tumor cells, and the ability to correlate the tumor shrinkage or growth with viral replication. Furthermore, much information about the precise mechanisms for how the virus attacks the tumor cells can be culled from this type of study. As more combination studies are performed, patterns in the clusters of genes involved will become apparent enabling researchers to pinpoint exactly how various viruses attack tumors and providing fruitful ideas for developing a new generation of recombinant viruses that are more effective.

## Author Contributions

All authors listed have made a substantial, direct, and intellectual contribution to the work and approved it for publication. AA drafted the article, reviewed the relevant literature, made substantial contributions to conception and design, interpreted the data and approved final version. JR drafted the article, reviewed the relevant literature, made substantial contributions to conception and design, interpreted the data, revised the article critically and approved final version. PZ made substantial contributions to conception and design, participated in revising it critically, interpreted the data, and approved final version. BG participated in revising it critically, interpreted the data, and approved final version.

## Conflict of Interest Statement

The authors declare that the research was conducted in the absence of any commercial or financial relationships that could be construed as a potential conflict of interest. The authors are all employees of Rapo Yerapeh Ltd.

## Abbreviations

ACC, adenoid cystic carcinoma; βhCG, beta-human chorionic gonadotropin; CEA, carcinoembryonic antigen; CDV, canine distemper virus; DAI, DNA-dependent activator of IFN− regulatory factors; DNA, deoxyribonucleic acid; ELISA, enzyme-linked immunosorbent assay; FDA, Food and Drug Administration; GBM, glioblastoma multiforme; GM-CSF, granulocyte-macrophage colony-stimulating factor; GnRH, gonadotropin releasing hormone; HSV, herpes simplex virus; IL, interleukin; IFN, interferon; IPA, Ingenuity Pathways Analysis; ISVP, infectious subviral particle; mRNA, messenger ribonucleic acid; MOI, multiplicity of infection; MV, measles virus; NDV, Newcastle disease virus; NOS/SCID, non-obese diabetic/severe combined immunodeficient; PCR, polymerase chain reaction; qPCR, quantitative polymerase chain reaction; RT-PCR, reverse transcription-polymerase chain reaction; ruc-GFP, Renilla luciferase-Aequorea-green fluorescent protein; SEA, superantigen *Staphylococcus* enterotoxin A; TNBC, triple negative breast cancer; TNF, tumor necrosis factor; VACV, vaccinia virus; VSV, vesicular stomatitis virus.
